# Vitamin D Status in Malaysian Men and Its Associated Factors

**DOI:** 10.3390/nu6125419

**Published:** 2014-11-26

**Authors:** Kok-Yong Chin, Soelaiman Ima-Nirwana, Suraya Ibrahim, Isa Naina Mohamed, Wan Zurinah Wan Ngah

**Affiliations:** 1 Department of Pharmacology, Universiti Kebangsaan Malaysia Medical Centre, Jalan Yaacob Latif, Bandar Tun Razak, Cheras, 56000 Kuala Lumpur, Malaysia; E-Mails: gabrielyong@live.com.my (K.-Y.C.); isanaina@yahoo.co.uk (I.N.M.); 2 Nutrition Science Program, School of Healthcare Sciences, Faculty of Health Sciences, Universiti Kebangsaan Malaysia, Jalan Raja Muda Abdul Aziz, 50300 Kuala Lumpur, Malaysia; E-Mail: surayagd@gmail.com; 3 Department of Biochemistry, Universiti Kebangsaan Malaysia Medical Centre, Jalan Yaacob Latif, Bandar Tun Razak, Cheras, 56000 Kuala Lumpur, Malaysia; E-Mail: zurina@medic.ukm.my

**Keywords:** men, osteoporosis, parathyroid, vitamin D

## Abstract

Vitamin D insufficiency is a global health problem. The data on vitamin D status in Malaysian men is insufficient. This study aimed to investigate vitamin D status among Chinese and Malay men in Malaysia and its associating factors. A cross-sectional study was conducted on 383 men aged 20 years and above, residing in Klang Valley, Malaysia. Their age, ethnicity, body anthropometry and calcaneal speed of sound (SOS) were recorded. Their fasting blood was collected for serum 25-hydroxyvitamin D (25(OH)D), intact parathyroid (PTH), total calcium and inorganic phosphate assays. Vitamin D deficiency was defined as a serum 25(OH)D level <30 nmol/L and insufficiency as a serum 25(OH)D level between 30 and 50 nmol/L. The overall prevalence of vitamin D deficiency was 0.5%, and insufficiency was 22.7%. Vitamin D deficiency and insufficiency were more prevalent in the Malays compared to the Chinese. Being Chinese, older in age, having lower body mass index (BMI) and a high physical activity status were associated significantly with a higher serum 25(OH)D level (*p* < 0.05). The serum PTH level was inversely associated with the serum 25(OH)D level (*p* < 0.05). As a conclusion, a significant proportion of Malaysian men have vitamin D insufficiency, although deficiency is uncommon. Steps should be taken to correct the vitamin D status of these men.

## 1. Introduction

Vitamin D is produced by our skin upon exposure to ultraviolet B (UVB) irradiation (280–320 nm) from sunlight. More than 90% of the circulating vitamin D in the body originates from the cutaneous production. Sunlight converts 7-dehydrocholesterol in the skin to vitamin D3, which will be transported to the liver and hydroxylated to 25-hydroxyvitamin D (25(OH)D). It will then be conveyed to the kidney and hydroxylated to 1,25-dihydroxyvitamin D (1,25(OH)D) [1,2]. Although 1,25(OH)D is the metabolically active prohormone, the 25(OH)D level is accepted as the determinant of vitamin D status [3]. Vitamin D deficiency will result in osteomalacia and rickets. Recent studies have uncovered that a low vitamin D level is associated with medical conditions, such as osteoporosis, metabolic disease, cancer, diabetes mellitus, infection and multiple sclerosis, but causality has not been proven for most outcomes [4].

The definitions of vitamin D deficiency and insufficiency are the subjects of much debate. Both the Institute of Medicine (IOM) of the United States and the Endocrine Society have come up with different definitions [5,6]. According to IOM, a serum 25(OH)D level above 50 nmol/L covers the requirement of at least 97.5% of the population [6]. This is in agreement with the statement by the World Health Organization, that the suppression of parathyroid hormone (PTH) with vitamin D supplementation only occurs in individuals with a baseline serum 25(OH)D level less than 50 nmol/L [7]. Thus, the current study adopted the cut-off value of 50 nmol/L for vitamin D insufficiency.

Vitamin D insufficiency is prevalent in countries with temperate climates that receive limited sunlight, especially during winter [8]. It is also prevalent in populations in the Middle East, whereby the covering of body parts is demanded by religious and cultural reasons [9,10,11]. Recent studies have demonstrated that vitamin D insufficiency is common in tropical countries, such as Vietnam, Malaysia and Indonesia [12,13,14]. In Malaysia, the studies on vitamin D status are limited to pockets of populations, such as school children [15], Malay employees of an academic institution [13], women of child-bearing age [12] and postmenopausal women [16]. There is a paucity of studies on men in this multiracial setting. Previous studies in the United States have revealed a significant racial discrepancy in vitamin D status among Caucasian, African American and Hispanic men [17,18]. We speculated that such a difference might occur in Malaysians.

The current study aimed to determine the prevalence of vitamin D status in Chinese and Malay adult men in Malaysia and its associating determinants. The relationships among serum 25(OH)D, PTH and bone health as reflected by calcaneal quantitative ultrasound (QUS) were also investigated. We hypothesized that there were significant relationships between ethnicity, age, physical activity and vitamin D status among Malaysian men. We also speculated that there was an inverse relationship between serum 25(OH)D and PTH and a positive relationship between 25(OH)D and bone health status. The information obtained from this study would identify risk factors for suboptimal vitamin D status in Malaysian men.

## 2. Experimental Section

### 2.1. Subjects

The subjects of this study were participants who attended the health screening session of the Malaysian Aging Male Study from September 2009 to September 2011 [19,20]. They were Chinese and Malay men aged 20 years and above living in Klang Valley (Kuala Lumpur and its environs). Purposive sampling was adopted, and solicitation was performed via radio broadcasts, newspapers, announcements at community centers and religious places. Subjects with the following conditions were excluded: (1) previously diagnosed with metabolic bone diseases, such as Paget’s disease, osteoporosis and osteomalacia; (2) previously diagnosed with conditions that might affect bone health, such as hyper/hypocalcaemia, hyper/hypoparathyroidism and renal failure; (3) undergoing treatment or taking medicine that might affect bone health, such as testosterone replacement/deprivation therapy, antiosteoporotic drugs, anticonvulsants, glucocorticoids, diuretics, thyroid supplements, lithium and thiazides; (4) had suffered a fracture or had undergone a major surgery within six months prior to the screening session; (5) had mobility problems and needed walking aids; and (6) taking vitamin D and/or calcium supplements. The subjects answered a questionnaire on their demographic details. The recording of medical history and physical examination were performed by qualified physicians. A total of 401 subjects consented to having blood taken and had enough serum for the 25(OH)D assay.

### 2.2. Research Ethics

The research protocol was reviewed and approved by the Research and Ethics Committee of Universiti Kebangsaan Malaysia Medical Centre (Code: AP-TKP-09-2009). Verbal and written explanation of the study was provided to the subjects in detail. Written consent was obtained before they were enrolled in the study.

### 2.3. Variables Collected

The age of the subjects was determined from the date of birth recorded on their identification cards. The ethnicity of the subjects was self-identified as Chinese, Malay, Indian or others.

The height of the subjects without shoes was measured with a Seca-213 portable stadiometer (SECA, Hamburg, Germany) and was recorded to the nearest 1 cm. The weight of the subjects with light clothing was determined with BC-418 Segmental Body Composition Analyzer (TANITA Corp., Tokyo, Japan) and was recorded to the nearest 0.1 kg. The body mass index was calculated as per the convention: body mass index (BMI) (kg/m^2^) = (body weight in kg)/(height^2^ in m^2^). The body fat percentage of the subjects was estimated with a BC-418 body composition analyzer using the bioelectrical impedance (BIA) method. The principles of BIA had been described elsewhere [21]. The waist circumference of the subjects was measured using a measuring tape midway between the lowest rib margin and the superior border of iliac crest at the end of a normal expiration in standing position. It was recorded to the nearest 1 cm.

The bone health of the subjects was determined using a CM-200 calcaneal ultrasonometer (Furuno Electric, Nishinomiya, Japan). The determinant generated was the calcaneal speed of sound (SOS) in meter per second (m/s). The right calcaneus of the subject was measured by a trained operator. Measurement was performed three times, and the average values were used in the analysis. Daily calibration was performed, and the device has a short-term *in vivo* precision error of 0.29% [21].

The blood of the subjects was collected in plain tubes by physicians or phlebotomists after an overnight fast of at least eight hours. The serum was extracted immediately and stored in −70 °C until being analyzed. The total calcium and inorganic phosphate levels were determined with an ADVIA 2400 auto-analyzer (Siemens Healthcare Diagnostics, Erlangen, Germany) using colorimetric methods. The serum intact parathyroid (PTH) (IBL International, Hamburg, Germany) and 25(OH)D (IDS, Tyne and Wear, U.K.) levels were determined using the enzyme-linked immunosorbent assay (ELISA), as per the manufacturer’s instructions. The interassays coefficient of variance values were 1.10%–1.17% for total calcium, 0.86%–1.19% for inorganic phosphate, 2.80%–3.60% for intact PTH and 4.60%–8.70% for 25(OH)D. Classification of vitamin D status was conducted as per the recommendation of IOM [6]. A serum 25(OH)D level lower than 30 nmol/L was considered as vitamin D deficiency, and a level between 30 and 50 nmol/L was considered as vitamin D insufficiency.

The physical activity of the subjects was determined using the self-administered International Physical Activity Questionnaire (IPAQ) (short form). The IPAQ was freely accessible online, and no permission was required to use it. Briefly, subjects were requested to recall the average amount of time spent in high-intensity activity, moderate-intensity activity, walking and sitting/lying down (except sleeping) in a week. The time spent, as recorded as number of days per week and minutes per day, was weighted by metabolic rates (METs) corresponding to the type of physical activity performed. Based on the results recorded in MET-minutes/week, the subjects were categorized as having high, moderate or low physical activity. The details of the assessment and classification of subjects based on their physical activity had been described elsewhere [21].

### 2.4. Statistical Analysis

The normality of the data was assessed using the Kolmogorov–Smirnov test. A square root transformation was performed on the 25(OH)D level, and a logarithmic transformation was performed on body weight and BMI to improve normality. The level of PTH and the IPAQ score were skewed and could not be improved via conventional transformation, so they were analyzed using a non-parametric test. The differences in characteristics between the Chinese and Malay men, as well as between subjects with optimal (≥50 nmol/L) and suboptimal (<50 nmol/L) serum 25(OH)D levels, were compared using the independent *t*-test for normally distributed data or the Mann–Whitney *U*-test for skewed data. The racial difference in the proportion of subjects in each vitamin D status was compared using the chi-squared test. The subjects were also divided according to age groups (10 years interval), and the serum 25(OH)D levels among the age groups were compared using one-way analysis of variance (ANOVA). The association between the variables was determined using multiple linear regression (MLR). Ethnicity (reference: Malays), PTH level (as in quartiles; reference: Q1) and physical activity status (as in “high”, “moderate” and “low”; reference: “low”) were coded as dummy variables and entered into the MLR. Continuous data were expressed as the mean and standard deviation (SD) for normally distributed data or as the median and interquartile range (IQR) for skewed data. Proportions were expressed as a percentage and the 95% confidence interval. The significant *p*-value was set at *p* < 0.05. Statistical analysis was performed using Statistical Package for Social Science (SPSS) version 21 (IBM, Armonk, USA).

## 3. Results

The serum 25(OH)D level was available for 401 subjects who attended the screening session. After eliminating univariate outliers, data from 383 subjects (60.8% Chinese and 39.2% Malay) were used in the current analysis. Data from 382 subjects were used in MLR analysis after the removal of one multivariate outlier. The Chinese subjects were significantly older, taller and had a lower BMI and physical activity level compared to the Malay subjects (*p* < 0.05). The Malay subjects also had significantly higher serum total calcium and inorganic phosphate levels, but a significantly lower serum 25(OH)D level compared to the Chinese subjects (*p* < 0.05) (Table 1).

**Table 1:** The difference in characteristics between Chinese and Malay subjects. IPAQ, International Physical Activity Questionnaire; MET, metabolic rate; n/a, not applicable; PTH, parathyroid.

Ethnicity	Chinese (*n* = 233)		Malays (*n* = 150)		*p*-value
Variable	Mean	SD	Mean	SD	
Age (years)	46.6	13.5	39.4	17.0	**<0.001**
Height (cm)	168.5	6.4	166.4	6.6	**0.002**
Body Fat Percentage (%)	21.9	5.7	22.9	7.0	0.130
Waist Circumference (cm)	87.5	8.7	89.0	12.3	0.206
Calcaneal Speed of Sound (m/s)	1516.5	26.7	1527.0	28.2	0.108 ^a^
Serum Total Calcium (mmol/L)	2.2	0.1	2.3	0.1	**<0.001**
Serum Inorganic Phosphate (mmol/L)	1.1	0.1	1.2	0.1	**<0.001**
**Variable**	**Median**	**IQR**	**Median**	**IQR**	***p*-value**
Body Weight (kg)	68.1	14.7	67.9	21.0	0.298
Body Mass Index (kg/m^2^)	24.0	5.1	24.8	7.7	**0.013**
Serum 25(OH)D Level (nmol/L)	60.2	13.9	54.6	16.0	**<0.001**
Serum PTH Level	43.4	22.0	42.8	20.2	0.609 ^b^
IPAQ Score (MET-min/week)	1099.0	2055.0	1,854.0	3,711.3	**<0.001 ^b^**
**Vitamin D Status**	***n***	**% (95% CI)**	***n***	**% (95% CI)**	***p*-value**
Deficiency	0/2	0	2/2	100	n/a
Insufficiency	36/85	42.4 (31.9–52.9)	49/85	57.7 (47.2–68.2)	0.159
Normal	197/296	66.6 (61.2–72.0)	99/296	33.5 (28.1–38.9)	**<0.001 ^c^**

All variables were tested using the independent *t*-test, unless indicated otherwise. ^a^ The calcaneal speed of sound is tested using univariate ANOVA adjusted for age and body mass index; ^b^ variables are tested using the Mann-Whitney *U*-test, because they are skewed and cannot be transformed; ^c^ the difference in proportions is analyzed using the chi-squared test. *p*-values in bold are significant.

**Figure 1:**
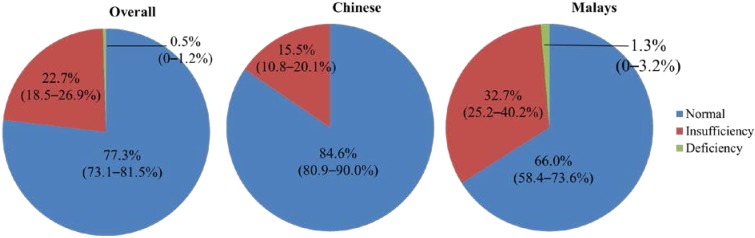
Vitamin D status of the subjects.

According to the definition by IOM, this study found that 22.7% of the overall subjects had vitamin D insufficiency and 0.5% suffered from deficiency. The two vitamin D-deficient subjects were Malay men. The proportion of subjects having vitamin D insufficiency was 15.5% for Chinese men and 32.7% for Malay men when segregated by ethnicity (Figure 1). Based on vitamin D status, there were significantly more Chinese subjects categorized as normal compared to Malay subjects (*p* < 0.05) (Table 1).

Subjects having a suboptimal serum 25(OH)D level (<50 nmol/L) were significantly younger, had a higher body weight, BMI, body fat percentage, waist circumference and serum inorganic phosphate level compared to subjects with an optimal 25(OH)D level (≥50 nmol) (*p* < 0.05) (Table 2).

**Table 2:** The difference in characteristics between subjects with an optimal (≥50 nmol) and suboptimal (<50 nmol) 25(OH)D level. PTH, parathyroid; IPAQ, International Physical Activity Questionnaire.

Vitamin D status	Subjects with 25(OH)D Level < 50 nmol (*n* = 87)		Subjects with 25(OH)D Level ≥ 50 nmol (*n* = 296)		*p*-value
Variable	Mean	SD	Mean	SD	
Age (years)	40.0	16.2	44.9	15.0	**0.009**
Height (cm)	167.2	6.2	167.8	6.6	0.519
Percentage of Body Fat (%)	23.8	6.4	21.8	6.1	**0.011**
Waist Circumference (cm)	90.2	10.6	87.5	10.1	**0.027**
Calcaneal Speed of Sound (m/s)	1522.0	29.2	1520.2	27.3	0.292 ^a^
Serum Total Calcium (mmol/L)	2.3	0.1	2.3	0.1	0.120
Serum Inorganic Phosphate (mmol/L)	1.1	0.1	1.1	0.1	**0.037**
**Variable**	**Median**	**IQR**	**Median**	**IQR**	***p*-value**
Body Weight (kg)	69.9	20.0	67.0	16.4	**0.012**
Body Mass Index (kg/m^2^)	25.6	6.6	24.0	5.3	**0.002**
Serum 25(OH)D Level (nmol/L)	44.3	6.8	61.6	11.8	**<0.001**
Serum PTH Level	43.3	26.5	43.0	20.7	0.279 ^b^
IPAQ Score (MET-min/week)	1580.0	2284.0	1386.0	2730.0	0.465 ^b^

All variables are tested using the independent *t*-test, unless indicated otherwise. ^a^ Speed of sound (SOS) is tested using univariate ANOVA, adjusted for age and BMI; ^b^ variables are tested using the Mann-Whitney *U*-test, because they are skewed and cannot be transformed; *p*-values in bold are significant.

The serum vitamin D level was significantly lower in subjects aged 20–29 years compared to subjects aged 40–49, 50–59 and more than 60 years (*p* < 0.05). Correspondingly, more subjects were categorized as having vitamin D deficiency and insufficiency in the younger age groups (Table 3).

Using stepwise MLR, BMI (β = −0.136; *p* = 0.007) was chosen as the best predictor of serum 25(OH)D level among the obesity indicators measured (waist circumference and body fat percentage were excluded), along with age (β = 0.150; *p* = 0.003), ethnicity β = 0.205, (Chinese *versus* Malays (reference); *p* < 0.001) and physical activity (β = 0.131, high *versus* low (reference) physical activity; *p* = 0.034). Being Chinese, older in age, having a lower BMI and a high physical activity status were significantly associated with a higher serum 25(OH)D level (*p* < 0.05) (Table 4).

**Table 3:** Serum 25(OH)D level and vitamin D status of the subjects in each age group.

Age Group (Years)	*N*	Serum 25(OH)D Level (nmol/L)		Vitamin D Status (%)		
		Mean	SD	Deficiency	Insufficiency	Sufficiency
20–29	91	54.7	13.1	1.1	33.0	65.9
30–39	58	58.3	12.0	1.7	22.4	75.9
40–49	78	60.3 ^a^	11.1	0.0	15.4	84.6
50–59	93	60.4 ^a^	12.1	0.0	20.4	79.6
≥60	63	60.2 ^a^	11.8	0.0	17.5	82.5
Overall	383	58.7	12.2	0.5	22.2	77.3

^a^ indicates a significant difference compared to the age group of 20–29 years, as indicated by one-way ANOVA.

**Table 4:** The association between the serum 25(OH)D level and its predictors. BMI, body mass index; PA, physical activity.

Variables	Standardized Regression	*p*-value
Ethnicity (Chinese *vs.* Malays (reference))	0.205	**<0.001**
Age	0.150	**0.003**
BMI	−0.136	**0.007**
Low PA *vs.* Moderate PA	−0.090	0.142
Low PA *vs.* High PA	0.131	**0.034**

*p*-values in bold are significant; *N* = 382 after elimination of a multivariate outlier.

**Figure 2:**
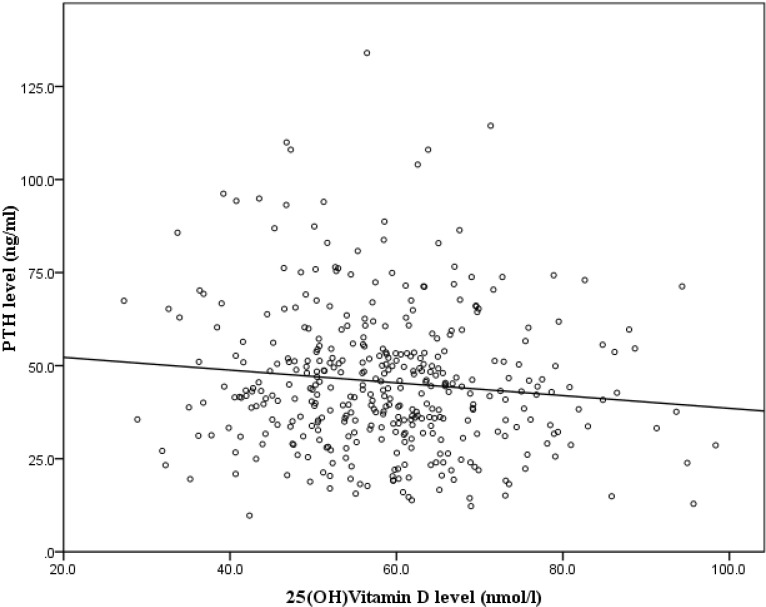
Scatter plot of the parathyroid (PTH) level *versus* the 25(OH)D level in the serum.

The associations between serum 25(OH)D and serum intact PTH, total calcium and inorganic phosphate levels were investigated using MLR. It was found that the difference between the lowest and the highest quartiles of the serum PTH level was associated with the variation of the serum 25(OH)D (β = −0.139, *p* = 0.025) level. Subjects in the lowest quartile of PTH level had a significantly higher serum 25(OH)D level compared to subjects in the highest quartiles of PTH level (*p* < 0.05). A plateau was not detected when the serum PTH level was plotted against the 25(OH)D level (Figure 2). However, there was no significant association between the serum 25(OH)D level and serum total calcium and inorganic phosphate levels (*p* > 0.05) (Table 5).

**Table 5:** The association between the serum 25(OH)D level and biochemical factors. PTH, parathyroid; IPAQ, International Physical Activity Questionnaire.

Variable	Standardized Regression	*p*-value
Serum Calcium	−0.018	0.737
Serum Inorganic Phosphate	0.009	0.861
PTH Q1 *vs*. Q2	−0.063	0.300
PTH Q1 *vs*. Q3	−0.068	0.269
PTH Q1 *vs*. Q4	−0.139	**0.025**

*p*-values in bold are significant; *N* = 382 after elimination of a multivariate outlier.

The association between calcaneal SOS value and biochemical determinants of calcium homeostasis (serum 25(OH)D, serum intact PTH, serum total calcium and serum inorganic phosphate levels) was studied. None of the predictors were significantly associated with SOS values after adjustment for age, BMI and ethnicity of the subjects (*p* > 0.05) (Table 6).

**Table 6:** The association between calcaneal speed of sound and biochemical variables related to calcium homeostasis. PTH, parathyroid.

Variables	Standardized Regression	*p*-value
PTH Q1 *vs*. Q2	−0.083	0.152
PTH Q1 *vs*. Q3	−0.024	0.687
PTH Q1 *vs*. Q4	−0.022	0.706
25(OH)D level	0.067	0.171
Serum Total Calcium (mmol/L)	0.020	0.705
Serum Inorganic Phosphate (mmol/L)	−0.001	0.979

The association has been adjusted for age, body mass index and ethnicity; *N* = 382 after elimination of a multivariate outlier.

## 4. Discussion

The current study found that vitamin D deficiency was uncommon in Malaysian men, whereby it only occurred in 0.5% of the study population. However, a significant proportion of the study population suffered from vitamin D insufficiency (32.7% Malay men and 15.5% Chinese men). Being Chinese, older in age, having a high physical activity level and a lower BMI were associated with a higher serum 25(OH)D level in Malaysian men. Serum PTH was inversely and significantly associated with the serum 25(OH)D level in the subjects. However, none of the serum biochemical markers (PTH, total calcium, inorganic phosphate, 25(OH)D) were associated with the bone health of the subjects, as reflected by calcaneal SOS.

The definitions of vitamin D deficiency and insufficiency put forward by the Endocrine Society and IOM were significantly different. While IOM defined deficiency as a serum 25(OH)D level less than 30 nmol/L and insufficiency as between 30 and 50 nmol/L [6], the cut-off values set by the Endocrine Society were significantly higher (deficiency: less than 50 nmol/L; insufficiency: 50–74 nmol/L) [5]. The use of different cut-off values would alter the prevalence of deficiency and insufficiency significantly. The arguments on the scientific merits of these definitions were presented elsewhere [22,23]. The IOM definitions were adopted in this study because previous studies showed that there was a congruence of bone beneficial effects at a serum 25(OH)D level between 40 and 50 nmol/L, but not higher [22].

The proportion of insufficiency reported in this study (22.2%) was considerably lower compared to previous studies. Moy and Bulgiba (2011) reported that the prevalence of vitamin D insufficiency (<50 nmol/L) in Malaysian Malay adult men (mean age: 49.6 ± 5.7 years) was 41.4% [13]. However, their subjects might not be representative of the Malay population, because they were limited to employees within a university campus. Hawkins (2013) found that in the Singaporean population (median age: 36 years (range: 19–71)), 30% of Chinese and 48% of Malay men had vitamin D insufficiency (<50 nmol/L) [24]. None of the subjects were found to have vitamin D deficiency (<30 nmol/L) [24]. It should be noted that the sample size in his study was small (40 men per ethnicity). In contrast to these studies, the current study recruited a larger sample size of free living Malaysian Chinese and Malay men. Thus, the subjects were more reflective of the population.

Racial discrepancy in vitamin D status as observed in this study had been found in other multiracial populations. Forrest and Stuhldreher (2011) reported that suboptimal vitamin D level (<50 nmol/L) was most commonly found in African Americans, followed by Hispanics and non-Hispanic whites in the United States aged 20 years and above [17]. In this study, the Malay men had a lower serum 25(OH)D level compared to the Chinese men. The darker pigmentation of the Malay men could have contributed to this observation. Previous studies had established that subjects with darker skin pigmentation had a lower 25(OH)D level after UVB radiation exposure [25,26]. This was because melanin absorbed and competed with 7-dehydrocholesterol for UVB photons [27]. This explanation on the discrepancy of vitamin D status between the Chinese and the Malay men remained speculative, because their skin pigmentation was not quantified in this study. However, other factors were less possible, because racial differences in clothing and adiposity were not apparent. The Malay subjects were significantly younger and more physically active, yet they had a significantly lower 25(OH)D level compared to the Chinese subjects.

The serum 25(OH)D level was found to be associated positively and significantly with age in this study. Previous studies on the association between age and serum 25(OH)D level were inconclusive. Several studies found that serum 25(OH)D level decreased with increasing age in men [9,10,28]. This complied with the observation that aging decreased the capacity of the skin to produce 7-dehydrocholesterol [29]. However, there were also studies showing that younger male subjects had a lower serum 25(OH)D level compared to older subjects [13,14,30,31]. This was attributed to lifestyle and behavioral changes across generations, such as a tendency to work indoors and avoidance of sunlight exposure in the younger generation. The exact reason for the positive association between serum 25(OH)D and age in this study was not known.

Body mass index was associated negatively with the serum 25(OH)D level of the subjects in this study. This was a universal finding across different populations [9,10,13,17,30,32]. The percentage of body fat and waist circumference were not significant predictors of serum 25(OH)D level. This was different from the observations of several studies, whereby a significant and negative relationship was found between serum 25(OH)D and waist circumference [9,10,13,33]. The prevailing explanation for the inverse relationship between vitamin D and obesity was that vitamin D, a fat-soluble vitamin, was sequestered in the adipose tissue [34]. A prospective study also reported that a lower vitamin D level was associated with a higher risk of developing metabolic syndrome, with obesity being one of the components [13,33]. As the current study was cross-sectional, it was impossible to determine the causal relationship between obesity as indicated by BMI and serum 25(OH)D in the subjects.

Physical activity status was found to be significantly and positively associated with the serum 25(OH)D level in this study. The association was more prominent in subjects with a high physical activity status compared to subjects with low physical activity status. Similarly, previous studies also demonstrated that physical activity, as determined using various different tools, was associated with vitamin D level [10,32,35]. It was speculated that subjects who were physically active also tended to spend more time under the sun. However, sunlight exposure was not quantified in this study. We also did not discriminate between outdoor and indoor physical activity. Previous studies had established that outdoor physical activity was more relevant to the vitamin D status of the subject [35,36].

The relationship between serum 25(OH)D and intact PTH level was significant and negative. This inverse association was more prominent between the lowest and the highest quartile of PTH. Previous studies showed that the relationship between 25(OH)D and PTH was inverse and linear [10,14]. Others found a transition point, whereby a further decrease of vitamin D caused an accelerated increase in PTH level [37,38]. However, this was not observed in the current study, probably because there were few subjects with a very low 25(OH)D level. The variation in serum 25(OH)D was not associated with serum total calcium and inorganic phosphate level. This was contributed to by the fact that serum calcium and inorganic phosphate levels were tightly regulated in the body and none of our subjects had medical conditions that could affect calcium and phosphate homeostasis. Furthermore, blood collection was performed after an overnight fast; thus, individual variation was minimized.

The bone health of men as determined by calcaneal SOS was not associated with the serum 25(OH)D level. This was different from previous studies, which found a significant relationship between the 25(OH)D level and bone health, indicated by bone mineral density (BMD) or QUS indices [39,40]. This discrepancy was probably because the subjects were generally healthy, and their serum 25(OH)D level did not explain much of the variation in their bone health status. On the other hand, there were more important factors contributing to bone health in these subjects, such as age [41], BMI [21], physical activity [21], testosterone [42,43] and sex hormone-binding globulin [42].

Several limitations should be considered when interpreting the results of the current study. The study population was limited to Chinese and Malay men in Klang Valley, Malaysia. Indian men were not recruited, due to logistical difficulties. The results should not be generalized to the whole Malaysian population. However, Chinese and Malay are the two largest ethnic groups in Klang Valley and in Malaysia [44]. Besides, men who visited the health screening session might be more health conscious and healthier. The bone health of the subjects was determined using a calcaneal QUS device, which generated SOS as the single determinant. Another QUS index, broadband ultrasound attenuation, which was shown to be associated with hip fracture risk independent of BMD [45], could not be generated by this device. Calcaneal SOS had been shown to correlate strongly with BMD assessed using dual-X-ray absorptiometry [46]. On the other hand, this study filled the gap in the knowledge of the vitamin D status in Malaysian men and its correlates. It showed that Malay men and the younger generation should be given emphasis in preventing the progression of vitamin D insufficiency and its associated health problems.

## 5. Conclusions

Although vitamin D deficiency is uncommon in Malaysian men, a significant proportion of them are suffering from vitamin D insufficiency. Men of Malay ethnicity, of a younger age, having a higher BMI and a sedentary lifestyle are at higher risk of having a lower serum 25(OH)D level. Steps should be taken to prevent the progression of vitamin D insufficiency and its associated health problems. The serum 25(OH)D level is not associated with bone health in these subjects. However, a prospective study is more suitable in establishing the effects of vitamin D insufficiency on bone health.
